# A Mathematical Description of the Flow in a Spherical Lymph Node

**DOI:** 10.1007/s11538-022-01103-6

**Published:** 2022-11-01

**Authors:** Giulia Giantesio, Alberto Girelli, Alessandro Musesti

**Affiliations:** 1 Dipartimento di Matematica e Fisica “N. Tartaglia”, Università Cattolica del Sacro Cuore, Brescia, Italy; 2 Dipartimento di Matematica e Applicazioni, Università degli Studi di Milano-Bicocca, Milan, Italy

**Keywords:** Lymph node, Darcy–Brinkman equation, Pulsatile flow, Spherical domain

## Abstract

The motion of the lymph has a very important role in the immune system, and it is influenced by the porosity of the lymph nodes: more than 90% takes the peripheral path without entering the lymphoid compartment. In this paper, we construct a mathematical model of a lymph node assumed to have a spherical geometry, where the subcapsular sinus is a thin spherical shell near the external wall of the lymph node and the core is a porous material describing the lymphoid compartment. For the mathematical formulation, we assume incompressibility and we use Stokes together with Darcy–Brinkman equation for the flow of the lymph. Thanks to the hypothesis of axisymmetric flow with respect to the azimuthal angle and the use of the stream function approach, we find an explicit solution for the fully developed pulsatile flow in terms of Gegenbauer polynomials. A selected set of plots is provided to show the trend of motion in the case of physiological parameters. Then, a finite element simulation is performed and it is compared with the explicit solution.

## Introduction

Lymph nodes are organs scattered throughout the lymphatic system which play a vital role in our immune response in breaking down bacteria, viruses and waste; the interstitial fluid (called *lymph* once inside the lymphatic system) is of fundamental importance in doing this since it transports these substances inside the lymph node (Arasa et al. 2021). The main features of the lymph node from a mechanical point of view are the presence of a porous bulk region (*lymphoid compartment*, LC), surrounded by a thin layer (*subcapsular sinus*, SCS) where the fluid can flow freely. More than 90% of the lymph remains in the SCS, while the remaining part enters into the LC through a conduit system network (Roozendaal et al. 2008; Grebennikov et al. 2016; Savinkov et al. 2017) formed by fibroblastic reticular cells (FRC), which is the *parenchyma* of the LN (Novkovic et al. 2020); due to this, LNs are organs with high resistance to flow. The bigger particles cannot enter the conduits formed by FRC and remain in the SCS, where they are confined and filtered by specialized cells of the lymphatic endothelial cells; however, there is some evidence that the selectivity of the FRC network is not based solely on the size of the molecules; indeed, selected macromolecules, such as antibodies, can gain access to the LN parenchyma (von Andrian and Mempel 2003).

Lymph flow inside LNs has an important function; indeed, fluid flow biases macromolecular distribution, enhances ligand expression, aligns extracellular matrix and shapes active mechanisms of cell migration. Fluid flow through endothelial monolayers and FRC networks enhances the expression of chemokines that direct leucocyte localization and migration patterns (O’Melia et al. 2019). Moreover, increased flows enhance proliferation and drug sensitivity in B cell lymphoma (Apoorva et al. 2018; Lamaison et al. 2020). Fluid flow is important to study the tumor metastasis (Birmingham et al. 2020) and drug transport (Permana et al. 2021). Despite its importance, as far as we know, only few models in the literature try to describe the behavior of the lymph from a mechanical point of view (Novkovic et al. 2018; Jafarnejad et al. 2015; Cooper et al. 2016, 2018; Tretiakova et al. 2021; Giantesio et al. 2021) or mimicking the LN mechanical properties in a LN-on-a-chip model (Shanti et al. 2020; Birmingham et al. 2020; Shanti et al. 2018).

In this paper, we propose a mathematical model for the flow of the interstitial fluid in a lymph node. We assume the lymph to be an incompressible fluid similar to water; moreover, we assume a small Reynolds number as a result of the small velocities within the lymph nodes (Moore and Bertram 2018), hence we can model the flow into the LC by Darcy–Brinkman equation [due to the high porosity and the time-dependence of the flow (Shanti et al. 2020; Savinkov et al. 2017)], and the flow inside the SCS by Stokes equation. The lymph enters the lymph node from the lymphatic vessels, which have a complex structure formed by one-way valves that prevent retrograde flow and a wall structure composed of sinus-lining cells: such cells control and generate active pulsation of the wall, pumping the lymph from a segment between two valves to another (the segment is called *lymphangion*) (Mozokhina and Savinkov 2020; Moore and Bertram 2018). This means that the lymph has a relevant pulsatile behavior, and we take it into account in our model. In Sect. 2 we describe the behavior of the lymph explicitly in spherical geometry, supposing that the fluid flow inside the lymph node is axisymmetric with respect to the azimuthal angle, so that we can assume a simplified two-dimensional geometry and we can use the stream function approach (Happel and Brenner 1983) to find an explicit solution. We remark that the solution given in Sect. 2.1 is quite general and can be used also for other choices of boundary conditions. Finally, in Sect. 3 we compare our results with some finite element simulations obtained using the open source software FreeFEM (Hecht 2012).

## Explicit Result in a Simplified Case

Let us model the lymph node (LN) as a spherical region: the subcapsular sinus (SCS) is a thin spherical shell with radii $R_1<R_2$ of creeping fluid flowing near the external wall of the LN, while the lymphoid compartment (LC) is a sphere of radius $R_1$ of porous material. We use spherical coordinates $(r,\theta ,\phi )$, where *r* is the radial distance, $\theta$ the polar angle and $\phi$ the azimuthal angle; moreover, we suppose axial symmetry with respect to the azimuthal angle $\phi$.

Assuming that the lymph, which flows inside the LN, is an incompressible fluid, and that the Reynolds number is small, we have the equations

1 $$\begin{aligned} \left\{ \begin{aligned}&\rho _0\dfrac{\partial \varvec{v}}{\partial t}(r,\theta ,t) = -\nabla p(r,\theta ,t) + \mu _e \Delta \varvec{v}(r,\theta ,t) - \dfrac{\mu }{k}\varvec{v}(r,\theta ,t){} & {} \quad r\in [0,R_1]\\&\rho _0\dfrac{\partial \varvec{v}}{\partial t}(r,\theta ,t) = -\nabla p(r,\theta ,t) + \mu \Delta \varvec{v}(r,\theta ,t){} & {} \quad r\in [R_1,R_2]\\&{\text {div}}\varvec{v}(r,\theta ,t)=0{} & {} \quad \end{aligned} \right. \nonumber \\ \end{aligned}$$

where $\rho _0$ is the constant density, $\varvec{v}$ the velocity, *p* is the pressure, $\mu$ the viscosity of the lymph, $\mu _e$ the *effective viscosity*, *k* the *permeability*. The second equation in (1) is the *Stokes equation* and describes the motion in the subcapsular sinus, the first is the *Darcy–Brinkman equation*, which is used for modeling the flow in the porous region of the LC, while the last equation models the incompressibility of the fluid. Here we assume a constant homogeneous permeability *k* (Savinkov et al. 2017; Shanti et al. 2020). The effective viscosity $\mu _e$ in general differs from the classical viscosity $\mu$ because $\mu _e$ keeps into account the Brinkman correction (Nield 2000). Furthermore, assuming that the flow is time periodic with period *T*, we write the time dependence of the velocity and of the pressure as a Fourier expansion

2 $$\begin{aligned} \varvec{v}(r,\theta ,t)=\sum _{m=- \infty }^{\infty }\varvec{v}_m (r,\theta )\text {e}^{i m \omega t}, \quad p(r,\theta ,t)=\sum _{m=- \infty }^{\infty }p_m (r,\theta )\text {e}^{i m \omega t}, \end{aligned}$$

where $$\omega =2\pi /T$$.

### Solving the Equations

Now we want to compute the general solution of system (1) in terms of the Fourier expansion (2). Here we try to be as general as possible, without imposing any boundary condition, so that out solution can be used in several situations. We will deal with suitable boundary conditions for our specific problem in Sect. 2.3.

By using (2), system (1) becomes

3 $$\begin{aligned} \left\{ \begin{aligned}&\Delta \varvec{v}_m(r,\theta )-\left( \dfrac{\mu }{k\mu _e}+\dfrac{im\omega \rho _0}{\mu _e}\right) \varvec{v}_m(r,\theta )=\dfrac{1}{\mu _e}\nabla p_m(r,\theta )&\quad \text {in } [0,R_1], \\&\Delta \varvec{v}_m(r,\theta )-\dfrac{im\omega \rho _0}{\mu }\varvec{v}_m(r,\theta )=\dfrac{1}{\mu }\nabla p_m(r,\theta )&\qquad \text {in } [R_1,R_2], \\&{\text {div}}\varvec{v}_m(r,\theta )=0,&\quad \end{aligned} \right. \end{aligned}$$

which can be written in compact form as

4 $$\begin{aligned} \left\{ \begin{aligned}&\Delta \varvec{v}_m(r,\theta )-q_m(r) \varvec{v}_m(r,\theta )=\dfrac{1}{\mu }\nabla p_m(r,\theta )\\&{\text {div}}\varvec{v}_m(r,\theta )=0, \end{aligned}\quad \qquad m\in \mathbb {Z}, \right. \end{aligned}$$

where $\mathbb {Z}$ is the set of integers, while $q_m$ is given by

5 $$\begin{aligned} q_m(r)={\left\{ \begin{array}{ll} \dfrac{\mu }{k\mu _e}+\dfrac{im\omega \rho _0}{\mu _e} &{} \quad \text {in } [0,R_1],\\ \dfrac{im\omega \rho _0}{\mu } &{} \quad \text {in } [R_1,R_2]. \end{array}\right. } \end{aligned}$$

Now, writing $\varvec{v}_m=v_{r,m}{{\textbf {e}}}_r+v_{\theta ,m}{{\textbf {e}}}_\theta$, we introduce the *stream function* $\psi _m$ (Happel and Brenner 1983) as

6 $$\begin{aligned} v_{r,m}(r,\theta )=-\dfrac{1}{r^2 \sin \theta }\dfrac{\partial \psi _m}{\partial \theta }, \quad v_{\theta ,m}(r,\theta )=\dfrac{1}{r \sin \theta }\dfrac{\partial \psi _m}{\partial r}. \end{aligned}$$

Moreover, it is useful to perform the change of variable $\zeta :=\cos \theta$, so that the previous equations become

7 $$\begin{aligned} v_{r,m}(r,\zeta )=\dfrac{1}{r^2}\dfrac{\partial \psi _m}{\partial \zeta },\quad v_{\theta ,m}(r,\zeta )=\dfrac{1}{r \sqrt{1-\zeta ^2}}\dfrac{\partial \psi _m}{\partial r}. \end{aligned}$$

By introducing the operator

$$\begin{aligned} {\text {E}}^{2}=\dfrac{\partial ^2}{\partial r^2}+ \dfrac{\left( 1-\zeta ^2\right) }{r^2}\dfrac{\partial ^2}{\partial \zeta ^2}, \end{aligned}$$

we can rewrite (4) as

8 $$\begin{aligned} {\text {E}}^{2}\left( {\text {E}}^{2}\psi _m(r,\zeta )\right) - q_m(r) {\text {E}}^{2}\psi _m(r,\zeta ) = 0,\quad m\in \mathbb {Z}, \end{aligned}$$

while for the pressure we have

9 $$\begin{aligned} {\left\{ \begin{array}{ll} \dfrac{\partial p_m}{\partial r}=\dfrac{\mu }{r^2} \dfrac{\partial }{\partial \zeta } \left( \left( {\text {E}}^{2}-q_m(r)\right) \psi _m\right) \\[2ex] \dfrac{\partial p_m}{\partial \zeta }=-\dfrac{\mu }{1-\zeta ^2}\dfrac{\partial }{\partial r}\left( \left( {\text {E}}^{2}-q_m(r)\right) \psi _m\right) \end{array}\right. }\qquad m\in \mathbb {Z}. \end{aligned}$$

Focusing on the case $m \ne 0$, we have that the solution can be written as

$$\begin{aligned} \psi _m(r,\zeta ) = \psi _{1,m}(r,\zeta ) + \psi _{2,m}(r,\zeta ), \end{aligned}$$

where

10 $$\begin{aligned} {\text {E}}^{2}\psi _{1,m}(r,\zeta )=0, \quad {\text {E}}^{2}\psi _{2,m}(r,\zeta )-q_m(r)\psi _{2,m}(r,\zeta )=0. \end{aligned}$$

We can now solve (10): by using the separation of variables

11 $$\begin{aligned} \psi _{1,m}(r,\zeta )=R(r)Z(\zeta ), \end{aligned}$$

substituting in the first equation of (10) we get

12 $$\begin{aligned} \dfrac{r^2}{R}\dfrac{\text {d}^2 R}{\text {d} r^2} + \dfrac{1-\zeta ^2}{Z}\dfrac{\text {d}^2 Z}{\text {d} \zeta ^2}=0. \end{aligned}$$

As the first term of (12) depends only on *r* and the second term only on $\zeta$, the two have to be constant, say $n(n-1)$ with $n \in \mathbb {N}$ (Haberman and Sayre 1958), where $\mathbb {N}$ is the set of natural numbers. Hence (12) becomes

13 $$\begin{aligned} r^2\dfrac{\text {d}^2 R}{\text {d} r^2} -n(n-1)R=0,\end{aligned}$$

14 $$\begin{aligned} \left( 1-\zeta ^2\right) \dfrac{\text {d}^2 Z}{\text {d} \zeta ^2} + n(n-1)Z=0. \end{aligned}$$

The solution of (13) is given by

15 $$\begin{aligned} R^{(n)}(r)=A^{(n)} r^n + B^{(n)} r^{1-n}, \end{aligned}$$

for some constants $A^{(n)},B^{(n)}$, while (14) is the *Gegenbauer equation*, whose solutions are the *Gegenbauer polynomials* $G_n,H_n$ with order $-1/2$, of the first and second kind, respectively. Hence the solution of the first equation of (10) becomes

$$\begin{aligned} \psi _{1,m}=\sum _{n=0}^{\infty }\left[ \left( A_m^{(n)} r^n + B_m^{(n)} r^{1-n}\right) G_n(\zeta )+\left( C_m^{(n)} r^n + D_m^{(n)} r^{1-n}\right) H_n(\zeta )\right] \end{aligned}$$

for some constants $A_m^{(n)},B_m^{(n)},C_m^{(n)},D_m^{(n)}$. Since $H_n$ is not smooth in $\zeta = \pm 1$ and $G_0$, $G_1$ lead to an infinite tangential velocity, the solution simplifies as

16 $$\begin{aligned} \psi _{1,m}(r,\zeta )=\sum _{n=2}^{\infty } \left( A_m^{(n)} r^n + B_m^{(n)} r^{1-n}\right) G_n(\zeta ), \end{aligned}$$

for some constants $A_m^{(n)},B_m^{(n)}$.

The second equation of (10) is

17 $$\begin{aligned} \dfrac{\partial ^2 \psi _{2,m}}{\partial r^2}+\dfrac{1-\zeta ^2}{r^2}\dfrac{\partial ^2 \psi _{2,m}}{\partial \zeta ^2}-q_m(r)\psi _{2,m}=0 \end{aligned}$$

and, using again the separation of variables,

$$\begin{aligned} \psi _{2,m}(r,\zeta )=R(r)Z(\zeta ), \end{aligned}$$

by a similar procedure as before, we obtain

18 $$\begin{aligned} \dfrac{\text {d}^2 R}{\text {d} r^2}-q_mR-\dfrac{n(n-1)}{r^2}R=0,\end{aligned}$$

19 $$\begin{aligned} (1-\zeta ^2)\dfrac{\text {d}^2 Z}{\text {d} \zeta ^2}+n(n-1)Z=0. \end{aligned}$$

Equation (18) is a Bessel equation, hence the solution can be written as

$$\begin{aligned} R^{(n)}(r)=\alpha ^{(n)} \sqrt{r}J_{n-\frac{1}{2}}\left( -i\sqrt{q_m}r\right) + \beta ^{(n)} \sqrt{r}Y_{n-\frac{1}{2}}\left( -i\sqrt{q_m}r\right) , \end{aligned}$$

where $J_s,Y_s$ are the *Bessel functions of the first and second kind*, respectively. Equation (19) is the same Gegenbauer equation as (14), hence the solution of (17) is given by

$$\begin{aligned} \psi _{2,m} =\sum _{n=2}^{\infty }\left[ \alpha _m^{(n)} \sqrt{r}J_{n-\frac{1}{2}}\left( -i\sqrt{q_m}r\right) +\beta _m^{(n)} \sqrt{r}Y_{n-\frac{1}{2}}\left( -i\sqrt{q_m}r\right) \right] G_n(\zeta ), \end{aligned}$$

and the general solution $\psi _m=\psi _{1,m}+\psi _{2,m}$ is

20 $$\begin{aligned} \psi _m(r,\zeta )= & {} \sum _{n=2}^{\infty }\Big [A_m^{(n)} r^n + B_m^{(n)} r^{1-n}+\alpha _m^{(n)} \sqrt{r}J_{n-\frac{1}{2}}\left( -i\sqrt{q_m}r\right) \nonumber \\{} & {} +\beta _m^{(n)} \sqrt{r}Y_{n-\frac{1}{2}}\left( -i\sqrt{q_m}r\right) \Big ] G_n(\zeta ). \end{aligned}$$

Now we want to employ the definition of $q_m$, so that we have to distinguish between the Stokes and the Darcy–Brinkman case. Let us denote with $A_m^{(n)}$, $B_m^{(n)}$, $\alpha _m^{(n)}$, $\beta _m^{(n)}$ the constants of the Stokes case ($R_1\le r\le R_2$) and with ${\bar{A}}_m^{(n)}$, ${\bar{B}}_m^{(n)}$, ${\bar{\alpha }}_m^{(n)}$, ${\bar{\beta }}_m^{(n)}$ those of the Darcy–Brinkman case ($0\le r\le R_1$). Using (5), we obtain, for any $m\ne 0$,

21 $$\begin{aligned} \psi ^S_m(r,\zeta )= & {} \sum _{n=2}^{\infty }\Bigg [A_m^{(n)} r^n + B_m^{(n)} r^{1-n}+\alpha _m^{(n)} \sqrt{r}J_{n-\frac{1}{2}}\left( -i\sqrt{\frac{i\rho _0m\omega }{\mu }}r\right) \nonumber \\{} & {} +\beta _m^{(n)} \sqrt{r}Y_{n-\frac{1}{2}}\left( -i\sqrt{\frac{i\rho _0m\omega }{\mu }}r\right) \Bigg ] G_n(\zeta ), \end{aligned}$$

22 $$\begin{aligned} \psi ^B_m(r,\zeta )= & {} \sum _{n=2}^{\infty }\left[ {\bar{A}}_m^{(n)}r ^n +{\bar{\alpha }}_m^{(n)}\sqrt{r}J_{n-\frac{1}{2}}\left( -i\sqrt{\dfrac{i\rho _0m\omega }{\mu _e}+\dfrac{\mu }{\mu _e k}}r\right) \right] G_n(\zeta ), \end{aligned}$$

where the superscript *S* denotes the Stokes case and *B* the Darcy–Brinkman case, and we used the fact that $r=0$ is in the domain of $\psi ^B$, so that ${\bar{B}}_m^{(n)}={\bar{\beta }}_m^{(n)}=0$ in view of the non degeneracy of the solution.

Regarding the pressure, we use (9) to obtain

23 $$\begin{aligned} p^S_m(r,\zeta )=C_m^S+im\omega \rho _0\sum _{n=2}^{\infty }\left[ \dfrac{A_m^{(n)}}{n-1}r^{n-1}-\dfrac{B_m^{(n)}}{n}r^{-n}\right] P_{n-1}(\zeta ) \end{aligned}$$

in the Stokes case, and

24 $$\begin{aligned} p^B_m(r,\zeta )=C_m^B+\left( im\omega \rho _0+\dfrac{\mu }{k}\right) \sum _{n=2}^{\infty }\dfrac{{\bar{A}}_m^{(n)}}{n-1}r^{n-1}P_{n-1}(\zeta ) \end{aligned}$$

in the Darcy–Brinkman case, where $P_n$ are the Legendre polynomials of the first kind.

For $m=0$ we get the well-known steady solution of the Stokes equation

25 $$\begin{aligned} \left\{ \begin{aligned}&\psi _0^S=\sum _{n=2}^{\infty }\left( A_0^{(n)}r^n+B_0^{(n)}r^{1-n} + C_0^{(n)}r^{n+2}+D_0^{(n)} r^{-n+3}\right) G_n(\zeta ),\\&p_0^S=C_0^S-\mu \sum _{n=2}^{\infty }\left[ \dfrac{2(2n+1)}{n-1}C_0^{(n)}r^{n-1}+ \dfrac{2(2n-3)}{n}D_0^{(n)} r^{-n}\right] P_{n-1}(\zeta ) \end{aligned} \right. \end{aligned}$$

and for the Darcy–Brinkman equation we have

26 $$\begin{aligned} \left\{ \begin{aligned}&\psi _0^B=\sum _{n=2}^{\infty }\left[ {\bar{A}}_0^{(n)}r^n+ {\bar{B}}_0^{(n)}\sqrt{r}J_{n-\frac{1}{2}} \left( -i\sqrt{\dfrac{\mu }{\mu _e k}}r\right) \right] G_n(\zeta ),\\&p_0^B=C_0^B+\dfrac{\mu }{k}\sum _{n=2}^{\infty } \left[ \dfrac{{\bar{A}}_0^{(n)}}{n-1}r^{n-1}\right] P_{n-1}(\zeta ). \end{aligned} \right. \end{aligned}$$

### Geometrical and Physiological Parameters

We use an idealized spherical geometry based on the data obtained from a murine (popliteal) lymph node: the radius is $R_2=0.5$ mm, the subcapsular sinus (SCS) thickness is $h=10\,\mu$m, the afferent and efferent lymphatic vessels have the same radius $R_{LV}=40 \, \mu$m (Birmingham et al. 2020; Kislitsyn et al. 2015; Ulvmar et al. 2014; Das et al. 2013; Shanti et al. 2020; Zhang et al. 2013; Jafarnejad et al. 2015). With these data, we have that more than $90 \%$ of the lymph takes the peripheral path without entering the LC in a pulsation cycle (Jafarnejad et al. 2015; Adair and Guyton 1983, 1985).

The inlet and outlet conditions are imposed in the upper and lower lymphatic vessel (near $\theta =0$ and $\theta =\pi$, respectively) as a pulsatile flow of the form

27 $$\begin{aligned} v_{\text {in}}(\theta ,t) = \frac{L}{\pi R_{LV}^2}f(t)H(\cos \theta ), \end{aligned}$$

where *L* is the maximum lymph mean flow of the inlet lymphatic vessel. Here we assume $L=10^{-3}\, \text {mm}^3$/s, as measured in (Blatter et al. 2016), and *f*(*t*) is a periodic function. The function *H* is given by

28 $$\begin{aligned} H(\zeta )={\left\{ \begin{array}{ll} \quad 1 &{}\quad \zeta \in [-1,-1+\zeta _0]\\ \quad 0 &{}\quad \zeta \in (-1+\zeta _0,1-\zeta _0)\\ -1 &{}\quad \zeta \in [1-\zeta _0,1], \end{array}\right. } \end{aligned}$$

where the constant $0<\zeta _0<1$ describes the inlet and outlet regions, and is given by

$$\begin{aligned} \zeta _0=\cos \left[ \arcsin \left( \frac{R_{LV}}{\sqrt{R_{LV}^2+R_2^2}}\right) \right] = \frac{R_2}{\sqrt{R_{LV}^2+R_2^2}}. \end{aligned}$$

Notice that we are assuming that the inlet and outlet velocities are the same.

The lymph is modeled as an incompressible Newtonian fluid similar to water (Moore and Bertram 2018) with viscosity $\mu =1$ mg/(mm s) and density $\rho _0 = 1$ mg/$\text {mm}^3$. The permeability is considered homogeneous (Savinkov et al. 2017) with value $k=3.84 \times 10^{-9} \, \text {mm}^2$ (Shanti et al. 2020). The effective viscosity is taken as $\mu _e=\frac{\mu }{\phi }$ (Ochoa-Tapia and Whitaker 1995a; Tan and Pillai 2009), where $\phi$ is the *porosity* taken as $\phi =0.75$ (Shanti et al. 2020). The parameters are summarized in Table 1.

**Table 1:** Physiological parameters of Sect. 2.2

Variable name	Value	Description
$R_2$	0.5 mm	External radius
*h*	$10 \, \upmu$m	Height of SCS
$R_1$	$R_2-h$	Internal radius
$R_{LV}$	$40 \, \upmu$m	Lymphatic vessel radius
$\mu$	1 mg/(mm s)	Viscosity
$\phi$	0.75	Porosity
$\mu _e$	$\frac{\mu }{\phi }$	Effective viscosity
$\rho$	1 mg/$\text {mm}^3$	Density
$\beta$	0.7	Stress jump
*k*	$3.84 \times 10^{-9} \, \text {mm}^2$	Permeability
*L*	$10^{-3} \, \text {mm}^3$/s	Maximum lymph fluid mean flow

### Boundary Conditions

We now want to impose suitable boundary conditions to our general solution. We give a *Dirichlet boundary condition* at the external boundary and the *Ochoa-Tapia boundary conditions* (Ochoa-Tapia and Whitaker 1995a, b) at the interface between the porous zone LC and the free-fluid region SCS. In this way we can close the problem and find a unique solution.

More precisely, we will assume the *no-slip condition* for the velocity on $R_2$, except near $\theta =0,\pi$, where we impose the inlet/outlet flow (27). For simplicity, given the small diameter of the afferent/efferent lymphatic vessel, we impose the inlet/outlet condition only for the radial velocity $v_r$, but we could use the same procedure to impose boundary condition for $v_{\theta }$ too. For the boundary conditions on the internal radius $R_1$, the Ochoa-Tapia boundary conditions imply the continuity of radial and tangential velocity, the continuity of the normal stress tensor and a jump-condition on the shear stress.

Thanks to the above conditions, we can determine for every *n* the six unknown constants in Eqs. (21)–(22). For the sake of brevity, we rewrite the stream functions as

$$\begin{aligned} \psi ^{S/B}_m(r,\zeta )=\sum _{n=2}^{\infty }{\tilde{\psi }}_{m,n}^{S/B}(r)G_n(\zeta ), \quad p_m^{S/B}=\sum _{n=2}^{\infty }{\tilde{p}}_{m,n}^{S/B}(r)P_{n-1}(\zeta ). \end{aligned}$$

Expanding the step function $H(\zeta )$ in (28) in terms of Legendre polynomials, we get

29 $$\begin{aligned} H(\zeta )=\sum _{n=2}^{\infty }b_{n-1}P_{n-1}(\zeta ), \end{aligned}$$

where

$$\begin{aligned} b_n=\frac{2n+1}{2}\int _{-1}^{1}H(\zeta )P_n(\zeta )\text {d}\zeta = \frac{2n+1}{2}\left( \int \limits _{-1}^{-1+\zeta _0}P_n(\zeta )\text {d}\zeta - \int \limits _{1-\zeta _0}^{1}P_n(\zeta )\text {d}\zeta \right) \end{aligned}$$

and we kept into account that $b_0=0$ since *H* is an odd function.

To impose the boundary condition, we need to expand in Fourier series the time dependence of (27), as we did in (2). Writing

$$\begin{aligned} f(t)=\sum _{m=-\infty }^{\infty }f_m\text {e}^{im\omega t}, \end{aligned}$$

it follows that

$$\begin{aligned} v_{\text {in}}(\zeta ,t)&=\frac{L}{\pi R_{LV}^2}H(\zeta )\sum _{m=-\infty }^{\infty }f_m\text {e}^{im\omega t}\\&=\frac{L}{\pi R_{LV}^2}\sum _{n=2}^{\infty }\sum _{m=-\infty }^{\infty }b_{n-1}P_{n-1}(\zeta )f_m\text {e}^{im\omega t}. \end{aligned}$$

Now we impose the boundary condition $v_r(R_2,\zeta ,t)=v_{\text {in}}(\zeta ,t)$: recalling the relation $G'_n(\zeta )=-P_{n-1}(\zeta )$, by (7)$_1$ we obtain

30 $$\begin{aligned} \dfrac{1}{R^2_2}\psi _m^S(R_2,\zeta )= -\frac{L}{\pi R_{LV}^2} \sum _{n=2}^{\infty }b_{n-1}G_n(\zeta ), \end{aligned}$$

whence

31 $$\begin{aligned} {\tilde{\psi }}_{m,n}^S(R_2)=-\frac{R_2^2 L}{\pi R_{LV}^2}b_{n-1}f_m \end{aligned}$$

for any $m\in \mathbb {Z}$ and $n\ge 2$, where we used the linear independence of the Gegenbauer polynomials.

By the no-slip boundary condition on $v_\theta$, recalling (7)$_2$ it follows that

32 $$\begin{aligned} \dfrac{\partial \psi _{m,n}^S}{\partial r}(R_2,\zeta )=0 \quad \Rightarrow \quad \dfrac{\partial {\tilde{\psi }}_{m,n}^S}{\partial r}(R_2)=0, \end{aligned}$$

where we used again the linear independence of the Gegenbauer polynomials.

We now write in terms of the stream function the Ochoa–Tapia boundary conditions on the internal radius $R_1$ (Prakash 2020), using the linear independence of Legendre and Gegenbauer polynomials:

- Continuity of $$v_r$$:
  33 $$\begin{aligned} v_{r,m}^S(R_1,\zeta )=v_{r,m}^B(R_1,\zeta ) \quad \Rightarrow \quad {\tilde{\psi }}_{m,n}^S(R_1)={\tilde{\psi }}_{m,n}^B(R_1). \end{aligned}$$
- Continuity of $$v_{\theta }$$:
  34 $$\begin{aligned} v_{\theta ,m}^S(R_1,\zeta )=v_{\theta ,m}^B(R_1,\zeta ) \quad \Rightarrow \quad \dfrac{\partial {\tilde{\psi }}_{m,n}^S}{\partial r}(R_1)=\dfrac{\partial {\tilde{\psi }}_{m,n}^B}{\partial r}(R_1). \end{aligned}$$
- Continuity of normal stress:
  $$\begin{aligned} {T}^S_{rr,m}(R_1,\zeta )={T}^B_{rr,m}(R_1,\zeta ), \end{aligned}$$
  where
  35 $$\begin{aligned} {T}_{rr,m}=-p_m+2\mu \dfrac{\partial v_{r,m}}{\partial r}; \end{aligned}$$
  we can write this condition as
  36 $$\begin{aligned} -{\tilde{p}}^S_{m,n}(R_1,\zeta )+4\mu \dfrac{1}{R^3_1}{\tilde{\psi }}_{m,n}^S(R_1,\zeta ) -2\mu \dfrac{1}{R_1^2}\dfrac{\partial {\tilde{\psi }}_{m,n}^S}{\partial r}(R_1,\zeta )=\\ =-{\tilde{p}}^B_{m,n}(R_1,\zeta )+4\mu _e\dfrac{1}{R^3_1}{\tilde{\psi }}_{m,n}^B(R_1,\zeta ) -2\mu _e\dfrac{1}{R_1^2}\dfrac{\partial {\tilde{\psi }}_{m,n}^B}{\partial r}(R_1,\zeta ). \end{aligned}$$
- The stress jump condition:
  37 $$\begin{aligned} {T}^S_{r\theta ,m}(R_1,\zeta )-{T}^B_{r\theta ,m}(R_1,\zeta )=\dfrac{\beta \mu }{\sqrt{k}}v^B_{\theta ,m}(R_1,\zeta ), \end{aligned}$$
  where $\beta$ is the *slip constant* which has to be estimated experimentally. Since the expression of the shear stress is
  38 $$\begin{aligned} {T}_{r\theta ,m}=\mu \left[ \dfrac{1}{r}\dfrac{\partial v_{r,m}}{\partial \theta }-\dfrac{v_{\theta ,m}}{r}+\dfrac{\partial v_{\theta ,m}}{\partial r}\right] , \end{aligned}$$
  in the term $\frac{\partial v_{r,m}}{\partial \theta }=-\sqrt{1-\zeta ^2}\frac{\partial v_{r,m}}{\partial \zeta }$ there is a second derivative of the Gegenbauer polynomials, so that we need the following property (Abramowitz and Stegun 1964; Zlatanovski 1999):
  39 $$\begin{aligned} G''_n(\zeta )=-\dfrac{n(n-1)}{1-\zeta ^2}G_n(\zeta ). \end{aligned}$$
  Hence we have:
  $$\begin{aligned} \dfrac{\partial v_{r,m}}{\partial \theta }= & {} -\sqrt{1-\zeta ^2}\dfrac{1}{r^2}\dfrac{\partial ^2 \psi _m}{\partial \zeta ^2}\\= & {} -\frac{\sqrt{1-\zeta ^2}}{r^2}\sum _{n=2}^{\infty }{\tilde{\psi }}_{m,n}(r) G''_n(\zeta ) =\frac{\sqrt{1-\zeta ^2}}{r^2}\sum _{n=2}^{\infty }n(n-1) {\tilde{\psi }}_{m,n}(r)G_n(\zeta ). \end{aligned}$$
  After some computations, Eq. (37) can be written as
  40 $$\begin{aligned}{} & {} \mu \left[ \dfrac{n(n-1)}{R_1^3}{\tilde{\psi }}_m^S(R_1)-\dfrac{2}{R_1^2}\dfrac{\partial {\tilde{\psi }}_{m,n}^S}{\partial r}(R_1)+\dfrac{1}{R_1}\dfrac{\partial ^2 {\tilde{\psi }}_{m,n}^S}{\partial r^2}(R_1)\right] \nonumber \\{} & {} \qquad -\mu _e\left[ \dfrac{n(n-1)}{R_1^3}{\tilde{\psi }}_{m,n}^B(R_1)-\dfrac{2}{R_1^2}\dfrac{\partial {\tilde{\psi }}_{m,n}^B}{\partial r}(R_1)+\dfrac{1}{R_1}\dfrac{\partial ^2 {\tilde{\psi }}_{m,n}^B}{\partial r^2}(R_1)\right] \nonumber \\{} & {} \quad =\dfrac{\beta \mu }{\sqrt{k}}\dfrac{1}{R_1}\dfrac{\partial {\tilde{\psi }}_{m,n}^B}{\partial r}(R_1). \end{aligned}$$

From (31)–(34), (36) and (40), for every $m\in \mathbb {Z}$ and $n\ge 2$ we obtain a linear system in the unknowns $(A_m^{(n)},B_m^{(n)},\alpha _m^{(n)},\beta _m^{(n)},{\bar{A}}_m^{(n)},{\bar{\alpha }}_m^{(n)})$, which are the constants of integration of Eqs. (21)–(22), and the same holds for the steady case when $m=0$ in the unknowns $(A_0^{(n)},B_0^{(n)},C_0^{(n)},D_0^{(n)},{\bar{A}}_0^{(n)},{\bar{B}}_0^{(n)})$ which are the constants of integration of Eqs. (25)$_1$–(26)$_1$.

Moreover, we fix the value of the pressure in one point to find the constants in equation (23)–(25) and have a physiological pressure value. By (36), it follows that $C_m^S=C_m^B$ and $C_0^B=C_0^S$. We fix the pressure (with respect to time) at the exit point $(r,\zeta )=(R_2,-1)$ by using the same time function of (27), that is,

$$\begin{aligned} p(t)={\bar{p}}f(t)={\bar{p}}\sum _{m=-\infty }^{\infty }f_m\text {e}^{im\omega t}. \end{aligned}$$

Hence we can find the pressure constants by imposing

$$\begin{aligned} p_m^S(R_2,-1)={\bar{p}}f_m,\quad m\in \mathbb {Z} \end{aligned}$$

where $p_m^S(r,\zeta )$ is given in (23) for $m\ne 0$, and in (25)$_2$ for $m=0$.

### Explicit Results

This section is devoted to show some plots related to the explicit solution and to make some considerations about the proposed model.

Following (Bertram et al. 2017), we choose a time function of the form

41 $$\begin{aligned} f(t)=\frac{1-\cos \pi t}{2}. \end{aligned}$$

We notice that in this case the period of a pulsatile flow in the lymph node is 2 s, hence $\omega =\pi$, and $f_m=0$ for $m\ne -1,0,1$.

In this model we do not take into account the inhibition and the autoregulation of the contractions in the lymphangion, given by several factors like shear stress and pressure (Bertram et al. 2019; Moore and Bertram 2018); a further extension of this model can be the coupling with a lymphangion model for taking into account these phenomena.

**Fig. 1:**
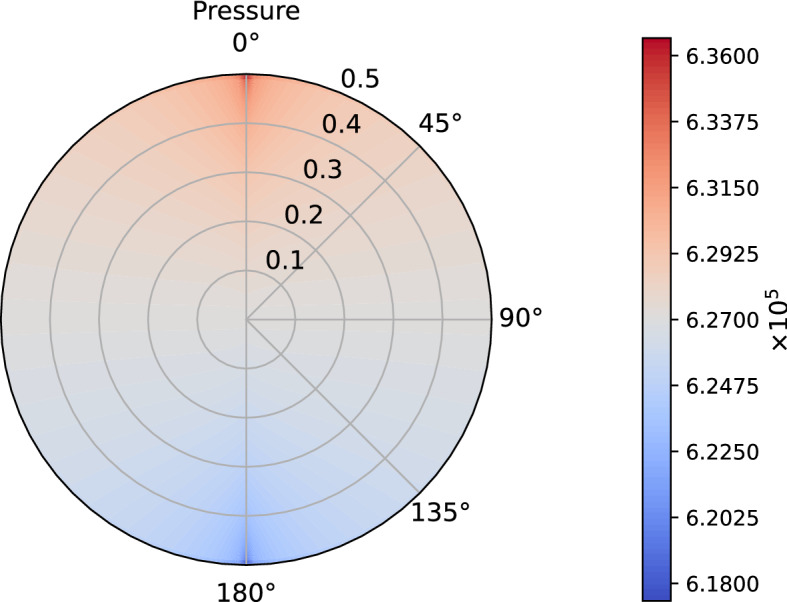
Pressure distribution in mPa with fixed pressure ${p}=6.18\times 10^5$ mPa at the outlet (color figure online)

In Fig. 1, we plot the pressure distribution in the LN with the fixed constant ${\bar{p}}=6.18\times 10^5$ mPa, corresponding to the lower limit of the pressure found in Bouta et al. (2014); as we can see, the values of the pressure belong to the range given in that paper and, due to the incompressibility of the flow, the pressure translates from a higher value in the inlet zone to a lower value in the outlet zone. We can choose to fix any pressure at the outlet, and we have the same pressure distribution with different values, for example with the fixed pressure of ${\bar{p}}=4\times 10^5$ mPa $\approx 3$ mmHg as in Jafarnejad et al. (2015).

**Fig. 2:**
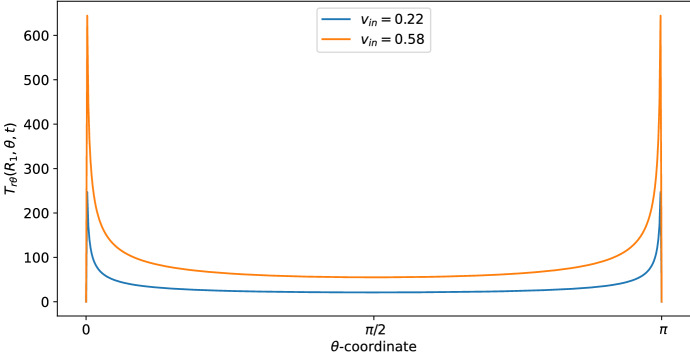
Shear stress $T_{r\theta }(r,\theta ,t)$ in mPa with respect to the polar angle ($\theta =0$ near the inlet flow and $\theta =\pi$ near the outlet flow) calculated at $t=1$ s and in the internal radius $R_1$ with different boundary velocities in mm/s (where $v_{\text {in}} \approx 0.22$ corresponds to $L=10^{-3}\, \text {mm}^3$/s and $v_{\text {in}}\approx 0.58$ corresponds to $L=2.2\times 10^{-3} \,\text {mm}^3$/s) (color figure online)

**Fig. 3:**
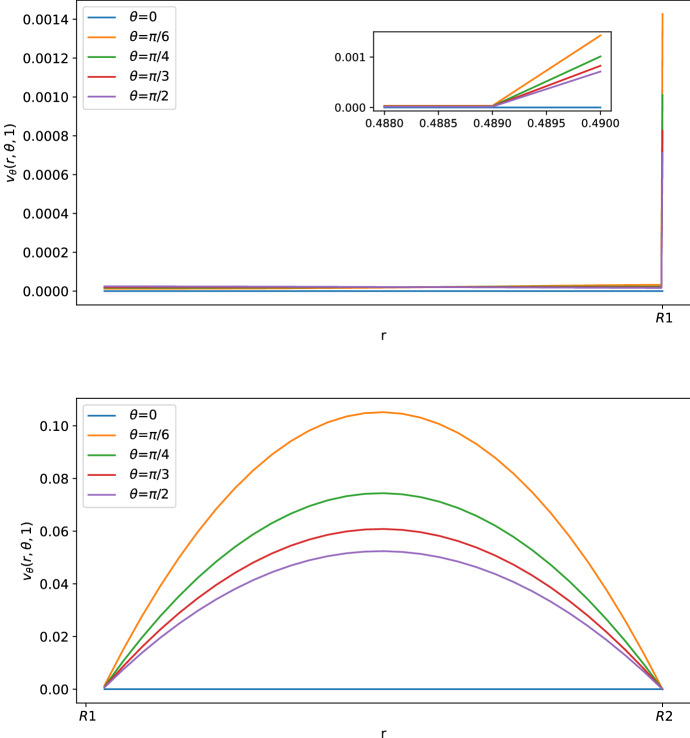
Tangential component of the velocity in mm/s with respect to the radius at different angles at $t=1$ s. The first picture corresponds to the tangential velocity in the LC (porous part), and the second corresponds to the tangential velocity in the SCS (free-fluid region) (color figure online)

Figure 2 provides the Stokes shear stress given by the formula:

$$\begin{aligned} T_{r\theta }=\sum _{m\in \{-1,0,1\}}\mu \left[ \dfrac{1}{r}\dfrac{\partial v_{r,m}}{\partial \theta }-\dfrac{v_{\theta ,m}}{r}+\dfrac{\partial v_{\theta ,m}}{\partial r}\right] \text {e}^{im\pi t}, \end{aligned}$$

(in mPa) at time $t=1$ s, where we have the maximum value of the velocity (and, consequently, of the shear stress) and radius $r=R_1$ (this is the shear stress at the exterior of the LC). We plot the shear stress value with two different boundary velocities: $v_{\text {in}}\approx 0.22$ corresponds to the physiological value of $L=10^{-3}\,\text {mm}^3$/s, given in Table 1, found in Blatter et al. (2016), and $v_{\text {in}} \approx 0.58$ appears in Jafarnejad et al. (2015). As we can see, the shear stress is similar to the one reported in Birmingham et al. (2020) and Jafarnejad et al. (2015); that is, higher near the inlet flow and lower near $\theta =\frac{\pi }{2}$. The same behavior occurs in the velocity too (see Fig. 3). This trend is interesting because the cell adhesion to the exterior of the LC is proportional to the shear stress (Birmingham et al. 2020), hence the majority of the cells adhere (and then enter in the LC) near the inlet zone of the lymphatic vessel. Indeed, in our model the inlet shear stress is the same as the outlet one due to the choice of the same inlet/outlet velocity and the incompressibility of the fluid; however, usually a part of the lymph enters in the blood capillaries in the LC (Adair and Guyton 1983, 1985), so that the shear stress in the outer zone reduces.

**Fig. 4:**
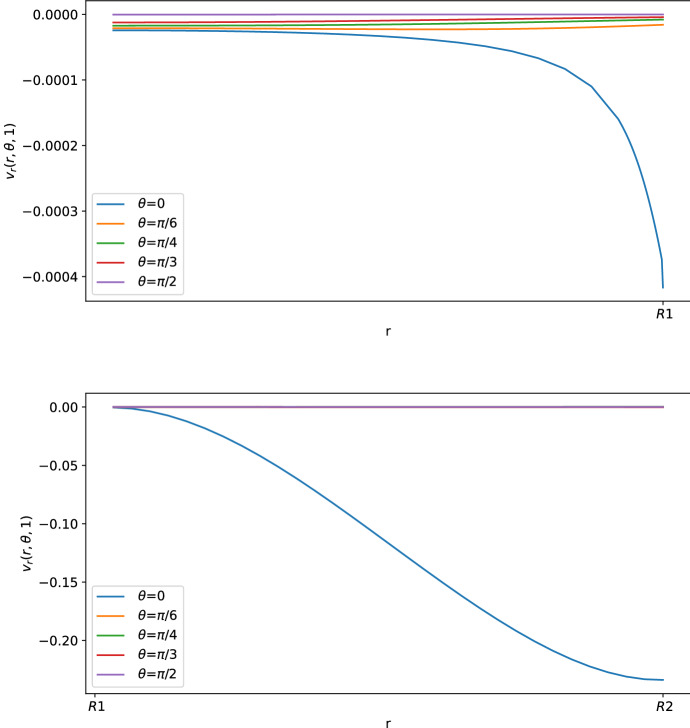
Normal component of the velocity in mm/s with respect to the radius at different angles at $t=1$ s (color figure online)

As we can see in Figs. 3 and 4, for $\theta >0$ the tangential component $\varvec{v}_\theta$ of the velocity in the SCS is the larger one. From the first picture in Fig. 3 one can see that the fluid flow in the porous medium is flat and starts increasing near the interface that connects the LC to the SCS, showing a non-differentiable point due to the Ochoa-Tapia boundary conditions (indeed, we do not impose the continuity of the derivative of $\varvec{v}_\theta$).

## Numerical Simulation

The explicit model found in the previous section uses several simplifications. In this section we propose some numerical simulations to describe a more general fluid flow in a lymph node.

We define two different domains and we call $\Omega ^S$ the domain of the SCS in which we have the Stokes equation, and $\Omega ^{B}$ the LC domain in which we have the Darcy–Brinkman equation. The boundaries of the domain are $\partial \Omega ^S=\Gamma ^S_D \cup \Gamma ^S_N$, where $\Gamma ^S_D$ is the part of the boundary with Dirichlet boundary condition and $\Gamma ^S_N$ is the one with the Neumann boundary condition and for the domain $\Omega ^B$ are $\partial \Omega ^B=\Gamma ^B_D \cup \Gamma ^B_N$, where $\Gamma ^B_D$ is the part of the boundary with Dirichlet boundary condition and $\Gamma ^B_N$ is the one with the Neumann boundary condition. We call the boundary interface of the two domains $\Gamma =\partial \Omega ^S \cap \partial \Omega ^B$. We define the normal $\varvec{n}$ at the interface $\Gamma$ as the external normal to $\Omega ^B$. Moreover, we define the spaces $W^I=\{\varvec{w}\in H^1 (\Omega ^I): \varvec{w}_{\Gamma _D}=0\}$, $W^I_g=\{ \varvec{v}\in H^1 (\Omega ^I): \varvec{v}_{\Gamma _D}=g\}$, $Q^I=\{q \in L^2(\Omega ^I),\ \text {with}\ \int _{\Omega ^I}q=0\ \text {if}\ \Gamma _D=\partial \Omega ^I\}$, where $I=S, B$.

The weak formulation of our problem is (supposing a constant density $\rho =\rho _0$ and viscosity $\nu =\mu /\rho _0$): find $\varvec{v}\in W_g^S$, $p \in Q^S$, $\varvec{v}_b \in W_g^B$ and $p_b \in Q^B$ such that

42 $$\begin{aligned}{} & {} \int _{\Omega ^S}\dfrac{\partial \varvec{v}}{\partial t}\cdot \varvec{w}\text {d}V - \dfrac{1}{\rho _0}\int _{\Omega ^S}p {\text {div}}\varvec{w}\text {d}V + \nu \int _{\Omega ^S}{{\textbf {D}}}(\varvec{v}): {{\textbf {D}}}(\varvec{w}) \text {d}V +\dfrac{1}{\rho _0}\int _{\Gamma ^S_N}{\textbf{T}}\varvec{w}\cdot \varvec{n}\text {d}S+\nonumber \\{} & {} \quad +\int _{\Omega ^B}\dfrac{\partial \varvec{v}_b}{\partial t}\cdot \varvec{w}_b\text {d}V - \dfrac{1}{\rho _0}\int _{\Omega ^B}p_b {\text {div}}\varvec{w}_b\text {d}V + \nu _e \int _{\Omega ^B}{{\textbf {D}}}(\varvec{v}_b): {{\textbf {D}}}(\varvec{w}_b)\text {d}V -\nonumber \\{} & {} \quad -\dfrac{1}{\rho _0}\int _{\Gamma ^B_N}{\textbf{T}}_e\varvec{w}_b\cdot \varvec{n}\text {d}S+\nu \int _{\Omega ^B}{\textbf{K}}^{-1}\varvec{v}_b\cdot \varvec{w}_b\text {d}V +\int _{\Omega ^S}{\text {div}}\varvec{v}q \text {d}V +\int _{\Omega ^B}{\text {div}}\varvec{v}_b q_b \text {d}V =0,\nonumber \\ \end{aligned}$$

for all $\varvec{w}\in W_g^S$, $\varvec{w}_b \in W_g^B$ such that $\varvec{w}=\varvec{w}_b \ \text {on} \ \Gamma$, and for all $q\in Q^S$ and $q_b \in Q^B$. In equation (42) we have that $\varvec{v}$ is the *velocity* in $\Omega ^S$, $p \in Q$ is the *pressure* in $\Omega ^S$, $\varvec{v}_b$ is the *velocity* in $\Omega ^B$, $p_b \in Q$ is the pressure in $\Omega ^B$, ${{\textbf {D}}}(\varvec{v})=1/2(\nabla \varvec{v}+ \nabla \varvec{v}^T)$, ${\textbf{T}}=-p{\textbf{I}}+\mu \left[ \nabla \varvec{v}+\nabla \varvec{v}^T\right]$, $\nu _e=\mu _e/\rho _0$, ${\textbf{K}}$ is the *permeability tensor* (in the case of Sect. 2, ${\textbf{K}}=k{\textbf{I}}$), ${\textbf{T}}_e=-p_b{\textbf{I}}+\mu _e\left[ \nabla \varvec{v}_b+\nabla \varvec{v}_b^T\right]$.

Now we want to write the weak formulation for the boundary condition 2.3; we have that the continuity of the velocity is verified automatically, and, for the stress-jump condition, we have (Tan and Pillai 2009) on the interface $\Gamma$:

$$\begin{aligned} \int _{\Gamma }{\textbf{T}}\varvec{w}\cdot \varvec{n}\text {d}S - \int _{\Gamma }{\textbf{T}}_e\varvec{w}\cdot \varvec{n}\text {d}S=\int _{\Gamma }\mu {{\textbf {B}}}\sqrt{{\textbf{K}}^{-1}} \varvec{v}_b \cdot \varvec{w}\text {d}S, \end{aligned}$$

where ${{\textbf {B}}}$ is the *slip tensor* (in the case of Sect. 2.3, ${{\textbf {B}}}=\beta {\textbf{I}}$).

The boundary conditions in the external wall (inlet condition and no-slip boundary condition) are imposed by the penalty method. Moreover, we add the Grad-div stabilization terms

$$\begin{aligned} \gamma _1\int _{\Omega ^{S/B}}{\text {div}}\varvec{v}\cdot {\text {div}}\varvec{w}\text {d}V+\gamma _2 \int _{\Omega ^{S/B}}{\text {div}}\left( \dfrac{\partial \varvec{v}}{\partial t}\right) \cdot {\text {div}}\varvec{w}\text {d}V \end{aligned}$$

in either Stokes and Darcy–Brinkman domains (Jenkins et al. 2014; Neilan and Zytoon 2020; Qin et al. 2020; Rong and Fiordilino 2020). Thanks to this stabilization term, we have the stability for the Darcy–Brinkman equation (see Xie et al. 2008). For the numerical discretization, we use a BDF2 method for the time discretization, instead, we use $\mathbb {P}^d_k - \mathbb {P}_k$ element pairs (where *k* is the polynomials order and *d* is the dimension) with the Brezzi-Pitkäranta stabilization, which consists in adding the term $\epsilon \int _{\Omega ^{S}}\nabla p \cdot \nabla q \text {d}V + \epsilon \int _{\Omega ^{B}}\nabla p_b \cdot \nabla q_b \text {d}V$ to the discretization of the Eq. (42), with $\epsilon \approx h_T^2$, where $h_T$ is the maximum diameter of the triangle of the finite element triangulation. The weak formulation here proposed has been implemented using the open source software FreeFEM.

### Numerical Test

In this section we want to qualitatively compare the results obtained with the numerical simulation with the explicit results exposed in Sect. 2. For that, we use the same geometry and parameters exhibited in Sect. 2.2; hence, in the external boundary we will impose only Dirichlet boundary condition ($\Gamma _N$ is empty), subdivided as $\Gamma _D=\Gamma _{\text {in}}\cup \Gamma _{\text {out}}\cup \Gamma _{\text {BC}}$, where we are imposing the inlet and the outlet flow in $\Gamma _{\text {in}}$ and $\Gamma _{\text {out}}$, respectively, given by the Eq. (27) with $L=10^{-3}\,\text {mm}^3$/s, and the no-slip boundary condition in $\Gamma _{\text {BC}}$. The numerical stabilization parameters are estimated as $\gamma _2=0$, while $\gamma _1=300$ in $\Omega ^S$ and $\gamma _1=10^6$ in $\Omega ^B$.

**Fig. 5:**
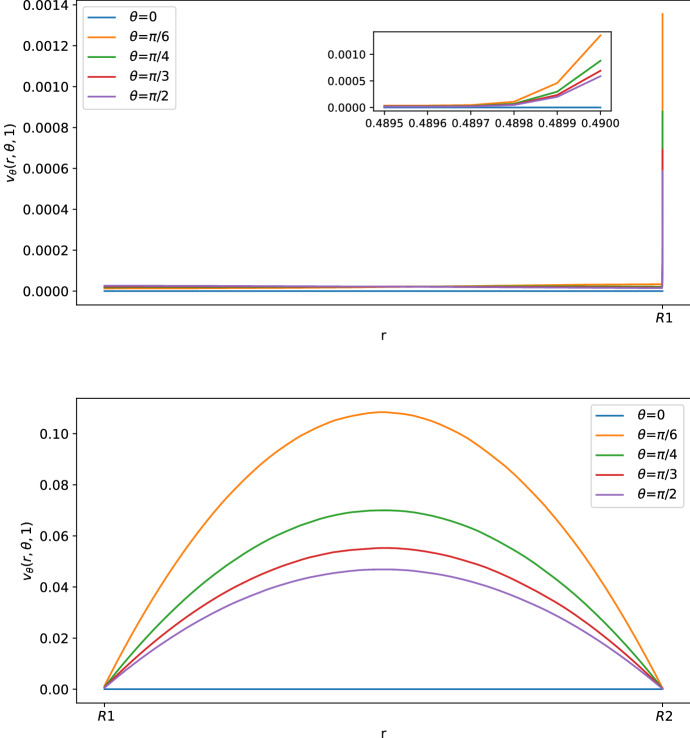
Tangential component of the velocity in mm/s with respect to the radius at different angles at $t=1$ s. The first graph corresponds to the tangential velocity in the LC (porous part), and the second corresponds to the tangential velocity in the SCS (free-fluid region) (color figure online)

**Fig. 6:**
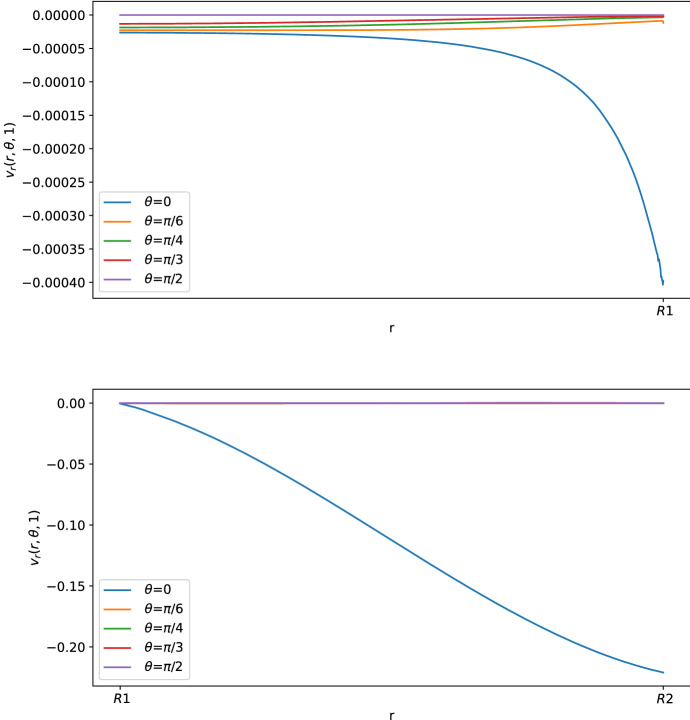
Normal component of the velocity in mm/s with respect to the radius at different angles at $t=1$ s. The first graph corresponds to the tangential velocity in the LC (porous part), and the second corresponds to the tangential velocity in the SCS (free-fluid region) (color figure online)

**Fig. 7:**
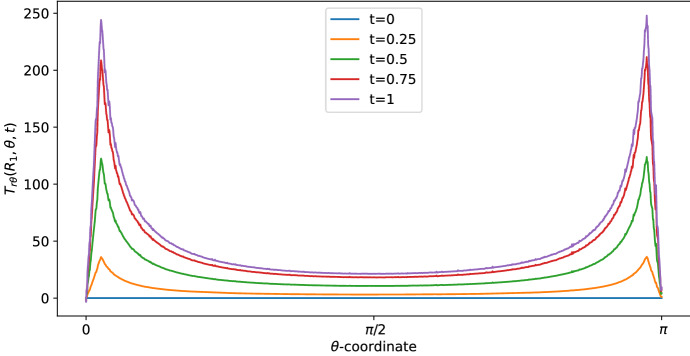
Shear stress in mPa with respect to the polar coordinates calculated at a fixed radius $r=R_1$ in different times (color figure online)

In Figs. 5, 6 and 7, we can see the tangential and radial velocity, and the shear stress, respectively. We can see that the results are very similar to the ones explicitly in Sect. 2.4: in order to remove some small oscillations in the internal velocity near $R_1$, we needed to use a finer mesh, which meant a greater computational cost for every time step. We can do only a qualitative comparison between the numerical solution of this section and the explicit solution in Sect. 2.4 because we do not have available and precise physiological data of the lymph node and we have an error in both cases: in the explicit result from the truncation of the sum, and here due to the finite element approximation. Qualitatively, we have the same behavior and values here and in the explicit result.

### Numerical Results

In this section we want to show a more complete numerical simulation using the method given in Sect. 3.

**Fig. 8:**
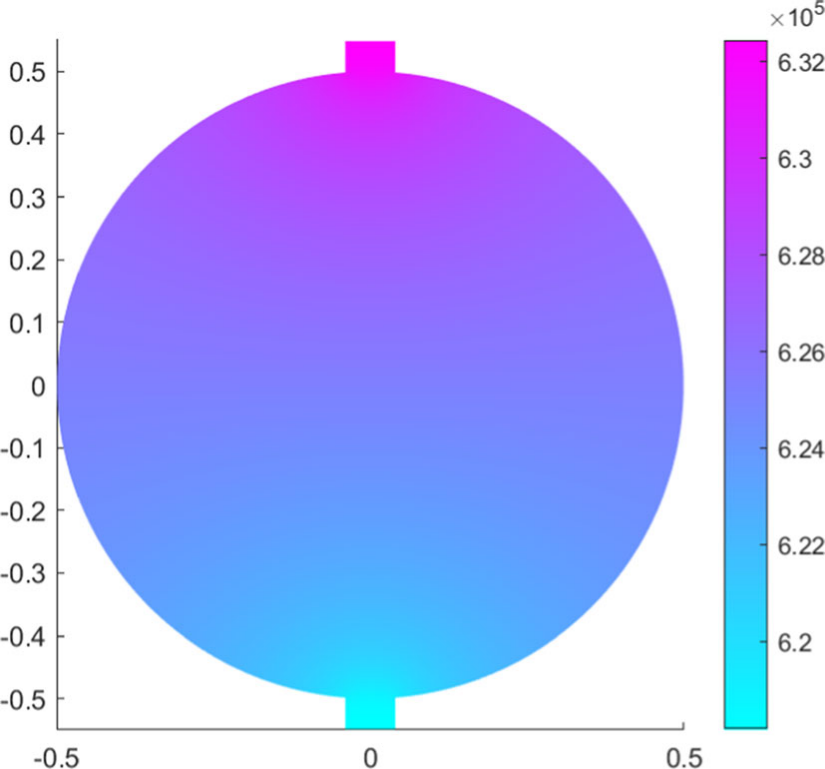
Pressure distribution in mPa with fixed pressure ${\bar{p}}=6.18\times 10^5$ mPa at outlet (color figure online)

We use a spherical idealized 2D geometry with the same parameters given in Sect. 2.2; hence we suppose that the permeability tensor ${\textbf{K}}$ is homogeneous and constant (this is not a limiting assumption, see Savinkov et al. (2017), Shanti et al. (2020), and Jafarnejad et al. (2015)) and the same with the slip tensor ${{\textbf {B}}}=\beta {\textbf{I}}$. Moreover, we add to the simulation domain a part to the inlet and outlet lymphatic vessel (see Figs. 10 and 8).

As we mention in Sect. 2.2, more than $90 \%$ of the lymph takes a peripheral path; the lymph that enters in the LC does not remain in the LC but gets out due to the incompressibility of the lymph, because we are not taking into account the fluid exchange behavior given by the blood vessels inside the LN.

The inlet condition is imposed in the upper lymphatic vessel as a uniform pulsatile flow in the *y* direction with the Eq. (27).

For the outlet condition, we need to fix the stress. For clarity and for a simpler interpretation, we fix the pressure ${\bar{p}}(t)$ in this way:

$$\begin{aligned} \left[ \int _{\Gamma ^S_N}{\textbf{T}}\varvec{w}\cdot \varvec{n}\text {d}S\right] _{\mid {\bar{p}}}&= \left[ \int _{\Gamma ^S_N}\left( -p{\textbf{I}}+\mu \left[ \nabla \varvec{v}+\nabla \varvec{v}^T\right] \right) \varvec{w}\cdot \varvec{n}\text {d}S\right] _{\mid {\bar{p}}}=\\&=\int _{\Gamma ^S_N}\left( -{\bar{p}}{\textbf{I}}+\mu \left[ \nabla \varvec{v}+\nabla \varvec{v}^T\right] \right) \varvec{w}\cdot \varvec{n}\text {d}S. \end{aligned}$$

We use the numerical parameters given in Sect. 3.1.

In Fig. 8, we can see the pressure distribution in the LN with ${\bar{p}}=6.18\times 10^5 f(t)$ mPa ($=6.3\,\text {cmH}_2\text {O}$ as the inferior limit in the range of pressure found in Bouta et al. (2014) and as in the explicit results in Sect. 2.4), where *f*(*t*) is the one given by the Eq. (41). As we can see, the pressure distribution is similar to the one in Fig. 1 and it is in range with the corresponding results. If one has ${\bar{p}}=4 \times 10^5 f(t)$ mPa ($=3$ mmHg as in Jafarnejad et al. (2015)), the behavior of the pressure is similar to the one showed in Fig. 8 (so that we omit the picture), with a range of values comparable to Jafarnejad et al. (2015).

**Fig. 9:**
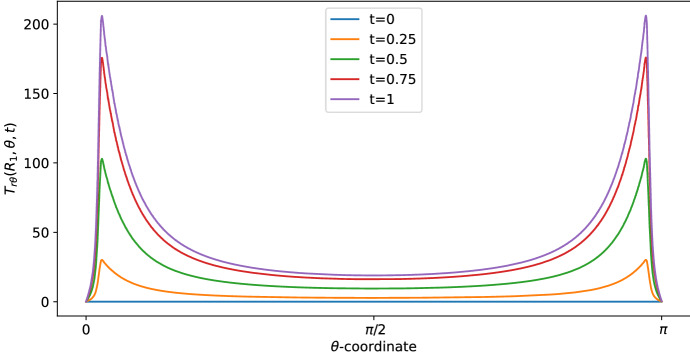
Shear stress in mPa with respect to the polar angle ($\theta =0$ near the inlet flow and $\theta =\pi$ near the outlet flow) calculated at different times (color figure online)

In Fig. 9, we can see the shear stress over time (in mPa). At time $t=1$ s, we have the maximum value of the velocity (and, consequently, of the shear stress) and the shear stress is similar to the one found in the explicit result (the blue curve with $v_{\text {in}} \approx 0.22$ plotted in Fig. 2), that is in range with the values found in Birmingham et al. (2020) and Jafarnejad et al. (2015).

**Fig. 10:**
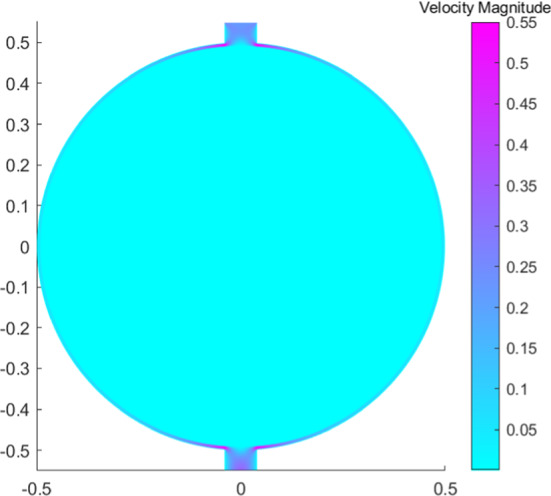
Velocity magnitude in mm/s at $t=1$ s (maximum velocity) (color figure online)

**Fig. 11:**
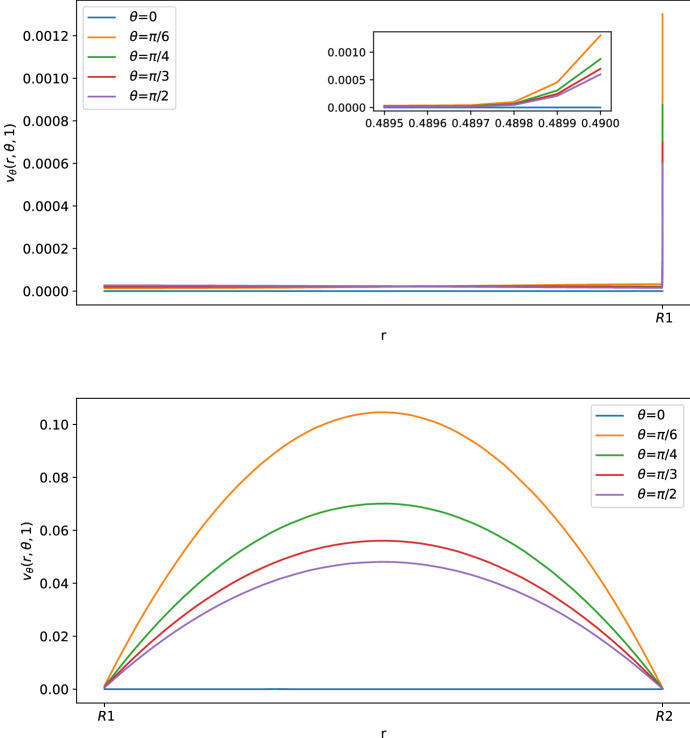
Tangential component of the velocity in mm/s with respect to the radius at different angles at $t=1$ s. The first graph corresponds to the tangential velocity in the LC (porous part), and the second corresponds to the tangential velocity in the SCS (free-fluid region) (color figure online)

We can see the norm and the velocity behavior in more details in Fig. 10. The tangential velocity (the most relevant one) is shown in Fig. 11. As expected, the maximum velocity is in the SCS near the inlet and the outlet region. In particular, we can see that the maximum velocity is between the connections of the SCS with the afferent/efferent vessel; then the velocity decrease with respect to the polar coordinate $\theta$, reaching the minimum at $\theta =\pi /2$. Moreover, even if we do not impose the outlet velocity equal to the inlet one, we have that this is true due to the incompressibility; hence our assumption used to find the explicit solution is not too limiting in this case.

## Conclusion

We proposed a model that describes the pulsatile lymph flow inside a simplified spherical lymph node (LN), using the Darcy–Brinkman equation to describe the lymph flow in the lymphoid compartment (LC, the porous part) and the Stokes equation to describe the flow inside the subcapsular sinus (SCS, the free fluid region). We found the explicit solution in terms of Gegenbauer polynomials and we showed the trend of the velocity, the pressure and the shear stress inside the LN; after that, we used this explicit solution to validate the numerical simulations of the model. Finally, we performed a more general numerical simulation with finite elements.

This model allows to better understand the fluid behavior inside the LN and how it changes with respect to time. The results obtained by our model are in agreement with the literature (Birmingham et al. 2020; Jafarnejad et al. 2015; Shanti et al. 2020; Cooper et al. 2016, 2018). We remark that the Ochoa-Tapia boundary condition minimally affects the fluid behavior in the SCS. Still, it affects the flow in the LC, inducing a velocity profile which is not smooth at the interface between the LC and SCS regions.

Particular attention was paid to the shear stress, because a lot of biological phenomena in the LN depend on it. Among them is the cell adhesion to the exterior of the LC, which is proportional to the shear stress: this is important because inside the LC there is a connection between the lymphatic system and the blood system, and some cells can get access from here to the blood circulation [for instance, tumor cells (Birmingham et al. 2020)]. Moreover, shear stress drives drug delivery and can affect pathologies like B-cell lymphoma (Apoorva et al. 2018; Lamaison et al. 2020). In our model we found that the shear stress is higher near the inlet and the outlet regions, and decreases with respect to the polar angle $\theta$, reaching the minimum at $\theta =\pi /2$; hence we believe that the majority of the cell adhesion is located near these two critical regions, which are the connections of the SCS with the afferent/efferent vessels.

Let us now make some considerations that can be interesting to improve the model in future. Here we have proposed to use a spherical geometry, but in general the LNs have a spheroidal shape (Giantesio et al. 2021; Jafarnejad et al. 2015; Cooper et al. 2016, 2018).

For simplicity, we did not take into account the fluid exchange inside the LN between the lymph in the fibroblastic reticular cells FRC and the blood in the capillaries, although it is important for the fluid regulation of the LN (Tobbia et al. 2009); a further extension of this work could take this phenomenon into account.

Moreover, in order to close our model and to find the unknown constants in the explicit solution, we used the Ochoa–Tapia boundary conditions, although other boundary conditions can be taken into consideration. For instance, a common choice is to impose the continuity also of the shear stress at the interface, which is tantamount to choose $\beta =0$ in Eq. (40). Using the same technique, one can address the more general conditions given in Angot et al. (2017) and Angot (2018), where there is a discontinuity also of the tangential velocity and the normal stress.

Another interesting and important extension of this model would be to couple the flow in the lymph node with the flow in the lymphangion, in order to have more realistic time pulsation and see how the lymph node affects and regulates the global lymph circulation (Bertram et al. 2017, 2019; Moore and Bertram 2018).

## Data Availability

Data sharing not applicable to this article as no datasets were generated or analysed during the current study.

## References

[CR1] Abramowitz M, Stegun IA (1964). Handbook of mathematical functions with formulas, graphs, and mathematical tables.

[CR2] Adair TH, Guyton AC (1983). Modification of lymph by lymph nodes. II. Effect of increased lymph node venous blood pressure. Am J Physiol Heart Circul Physiol.

[CR3] Adair TH, Guyton AC (1985). Modification of lymph by lymph nodes. III. Effect of increased lymph hydrostatic pressure. Am J Physiol Heart Circul Physiol.

[CR4] Angot P (2018). Well-posed Stokes/Brinkman and Stokes/Darcy coupling revisited with new jump interface conditions. ESAIM: Math Model Numer Anal.

[CR5] Angot P, Goyeau B, Ochoa-Tapia JA (2017). Asymptotic modeling of transport phenomena at the interface between a fluid and a porous layer: jump conditions. Phys Rev E.

[CR6] Apoorva F, Loiben AM, Shah SB, Purwada A, Fontan L, Goldstein R, Kirby BJ, Melnick AM, Cosgrove BD, Singh A (2018). How biophysical forces regulate human B cell lymphomas. Cell Rep.

[CR7] Arasa J, Collado-Diaz V, Halin C (2021). Structure and immune function of afferent lymphatics and their mechanistic contribution to dendritic cell and T cell trafficking. Cells.

[CR8] Bertram C, Macaskill C, Davis M, Moore J (2017). Valve-related modes of pump failure in collecting lymphatics: numerical and experimental investigation. Biomech Model Mechanobiol.

[CR9] Bertram C, Macaskill C, Moore J (2019). Inhibition of contraction strength and frequency by wall shear stress in a single-lymphangion model. J Biomech Eng.

[CR10] Birmingham KG, O’Melia MJ, Bordy S, Aguilar DR, El-Reyas B, Lesinski G, Thomas SN (2020). Lymph node subcapsular sinus microenvironment-on-a-chip modeling shear flow relevant to lymphatic metastasis and immune cell homing. Iscience.

[CR11] Blatter C, Meijer EF, Nam AS, Jones D, Bouma BE, Padera TP, Vakoc BJ (2016). In vivo label-free measurement of lymph flow velocity and volumetric flow rates using Doppler optical coherence tomography. Sci Rep.

[CR12] Bouta EM, Wood RW, Brown EB, Rahimi H, Ritchlin CT, Schwarz EM (2014). In vivo quantification of lymph viscosity and pressure in lymphatic vessels and draining lymph nodes of arthritic joints in mice. J Physiol.

[CR13] Cooper LJ, Heppell JP, Clough GF, Ganapathisubramani B, Roose T (2016). An image-based model of fluid flow through lymph nodes. Bull Math Biol.

[CR14] Cooper L, Zeller-Plumhoff B, Clough G, Ganapathisubramani B, Roose T (2018). Using high resolution X-ray computed tomography to create an image based model of a lymph node. J Theor Biol.

[CR15] Das S, Sarrou E, Podgrabinska S, Cassella M, Mungamuri SK, Feirt N, Gordon R, Nagi CS, Wang Y, Entenberg D (2013). Tumor cell entry into the lymph node is controlled by CCL1 chemokine expressed by lymph node lymphatic sinuses. J Exp Med.

[CR16] Giantesio G, Girelli A, Musesti A (2021). A model of the pulsatile fluid flow in the lymph node. Mech Res Commun.

[CR17] Grebennikov D, Van Loon R, Novkovic M, Onder L, Savinkov R, Sazonov I, Tretyakova R, Watson DJ, Bocharov G (2016). Critical issues in modelling lymph node physiology. Computation.

[CR18] Haberman WL, Sayre RM (1958) Motion of rigid and fluid spheres in stationary and moving liquids inside cylindrical tubes. Technical report, David Taylor Model Basin, Washington DC

[CR19] Happel J, Brenner H (1983). Low Reynolds number hydrodynamics: with special applications to particulate media.

[CR20] Hecht F (2012). New development in FreeFem++. J Numer Math.

[CR21] Jafarnejad M, Woodruff MC, Zawieja DC, Carroll MC, Moore J (2015). Modeling lymph flow and fluid exchange with blood vessels in lymph nodes. Lymphat Res Biol.

[CR22] Jenkins EW, John V, Linke A, Rebholz LG (2014). On the parameter choice in grad-div stabilization for the Stokes equations. Adv Comput Math.

[CR23] Kislitsyn A, Savinkov R, Novkovic M, Onder L, Bocharov G (2015). Computational approach to 3D modeling of the lymph node geometry. Computation.

[CR24] Lamaison C, Latour S, Hélaine N, Morvan VL, Monvoisin C, Mahouche I, Dussert C, Dessauge E, Pangault C, Seffals M, Broca-Brisson L, Alessandri K, Soubeyran P, Mourcin F, Nassoy P, Recher G, Tarte K, Bresson-Bepoldin L (2020). Stromal cells regulate malignant B-cell spatial organization, survival, and drug response in a new 3D model mimicking lymphoma tumor niche. bioRxiv.

[CR25] Moore JE, Bertram CD (2018). Lymphatic system flows. Annu Rev Fluid Mech.

[CR26] Mozokhina A, Savinkov R (2020). Mathematical modelling of the structure and function of the lymphatic system. Mathematics.

[CR27] Neilan M, Zytoon A (2020). Connection between grad-div stabilized Stokes finite elements and divergence-free Stokes finite elements. Int J Numer Anal Model.

[CR28] Nield DA (2000). Modelling fluid flow and heat transfer in a saturated porous medium. J Appl Math Decis Sci.

[CR29] Novkovic M, Onder L, Cheng H-W, Bocharov G, Ludewig B (2018). Integrative computational modeling of the lymph node stromal cell landscape. Front Immunol.

[CR30] Novkovic M, Onder L, Bocharov G, Ludewig B (2020). Topological structure and robustness of the lymph node conduit system. Cell Rep.

[CR31] Ochoa-Tapia JA, Whitaker S (1995). Momentum transfer at the boundary between a porous medium and a homogeneous fluid I. Theoretical development. Int J Heat Mass Transf.

[CR32] Ochoa-Tapia JA, Whitaker S (1995). Momentum transfer at the boundary between a porous medium and a homogeneous fluid. II Comparison with experiment. Int J Heat Mass Transf.

[CR33] O’Melia MJ, Lund AW, Thomas SN (2019). The biophysics of lymphatic transport: engineering tools and immunological consequences. Iscience.

[CR34] Permana AD, Nainu F, Moffatt K, Larrañeta E, Donnelly RF (2021). Recent advances in combination of microneedles and nanomedicines for lymphatic targeted drug delivery. Wiley Interdiscip Rev Nanomed Nanobiotechnol.

[CR35] Prakash J (2020). Hydrodynamic mobility of a porous spherical particle with variable permeability in a spherical cavity. Microsyst Technol.

[CR36] Qin Y, Hou Y, Huang P, Wang Y (2020). Numerical analysis of two grad-div stabilization methods for the time-dependent Stokes/Darcy model. Comput Math Appl.

[CR37] Rong Y, Fiordilino JA (2020). Numerical analysis of a BDF2 modular grad-div stabilization method for the Navier–Stokes equations. J Sci Comput.

[CR38] Roozendaal R, Mebius RE, Kraal G (2008). The conduit system of the lymph node. Int Immunol.

[CR39] Savinkov R, Kislitsyn A, Watson DJ, van Loon R, Sazonov I, Novkovic M, Onder L, Bocharov G (2017). Data-driven modelling of the FRC network for studying the fluid flow in the conduit system. Eng Appl Artif Intell.

[CR40] Shanti A, Teo J, Stefanini C (2018). In vitro immune organs-on-chip for drug development: a review. Pharmaceutics.

[CR41] Shanti A, Samara B, Abdullah A, Hallfors N, Accoto D, Sapudom J, Alatoom A, Teo J, Danti S, Stefanini C (2020). Multi-compartment 3D-cultured organ-on-a-chip: towards a biomimetic lymph node for drug development. Pharmaceutics.

[CR42] Tan H, Pillai KM (2009). Finite element implementation of stress-jump and stress-continuity conditions at porous-medium, clear-fluid interface. Comput Fluids.

[CR43] Tobbia D, Semple J, Baker A, Dumont D, Semple A, Johnston M (2009). Lymphedema development and lymphatic function following lymph node excision in sheep. J Vasc Res.

[CR44] Tretiakova R, Setukha A, Savinkov R, Grebennikov D, Bocharov G (2021). Mathematical modeling of lymph node drainage function by neural network. Mathematics.

[CR45] Ulvmar MH, Werth K, Braun A, Kelay P, Hub E, Eller K, Chan L, Lucas B, Novitzky-Basso I, Nakamura K (2014). The atypical chemokine receptor CCRL1 shapes functional CCL21 gradients in lymph nodes. Nat Immunol.

[CR46] Von Andrian UH, Mempel TR (2003). Homing and cellular traffic in lymph nodes. Nat Rev Immunol.

[CR47] Xie X, Xu J, Xue G (2008). Uniformly-stable finite element methods for Darcy–Stokes–Brinkman models. J Comput Math.

[CR48] Zhang Z, Procissi D, Li W, Kim D-H, Li K, Han G, Huan Y, Larson AC (2013). High resolution MRI for non-invasive mouse lymph node mapping. J Immunol Methods.

[CR49] Zlatanovski T (1999). Axisymmetric creeping flow past a porous prolate spheroidal particle using the Brinkman model. Q J Mech Appl Math.

